# Lifestyle-associated health risk indicators across a wide range of occupational groups: a cross-sectional analysis in 72,855 workers

**DOI:** 10.1186/s12889-020-09755-6

**Published:** 2020-11-04

**Authors:** Daniel Väisänen, Lena V. Kallings, Gunnar Andersson, Peter Wallin, Erik Hemmingsson, Elin Ekblom-Bak

**Affiliations:** 1 Department of Physical Activity and Health, The Swedish School of Sport and Health Sciences, Stockholm, Sweden; 2 HPI Health Profile Institute, Stockholm, Sweden

**Keywords:** Occupations, Risk indicators, Physical activity pattern, Cardiorespiratory fitness, Occupational groups, White-collar, Blue-collar, Lifestyle, Low- and high-skilled occupations

## Abstract

**Background:**

Identify and compare health risk indicators for common chronic diseases between different occupational groups.

**Methods:**

A total of 72,855 participants (41% women) participating in an occupational health service screening in 2014–2019 were included. Occupation was defined by the Swedish Standard Classification of Occupation, and divided into nine major and additionally eight sub-major groups. These were analysed separately, as white- and blue-collar occupations and as low- and high-skilled occupations. Seven health risk indicators were self-reported: exercise, physical work situation, sitting at work and leisure, smoking, diet, and perceived health, whereas cardiorespiratory fitness, BMI and blood pressure were measured. These were further dichotomized (yes/no) and as clustering of risk indicators (≥3 vs. <3).

**Results:**

The greatest variation in OR across sub-major and major occupational groups were seen for daily smoking (OR = 0.68 to OR = 5.12), physically demanding work (OR = 0.55 to OR = 45.74) and high sitting at work (OR = 0.04 to OR = 1.86). For clustering of health risk indicators, blue-collar workers had significantly higher clustering of health risks (OR: 1.80; 95% CI 1.71–1.90) compared to white-collar workers (reference). Compared to high-skilled white-collar workers, low-skilled white-collar workers had similar OR (2.00; 1.88–2.13) as high-skilled blue-collar workers (1.98; 1.86–2.12), with low-skilled blue-collar workers having the highest clustered risk (2.32; 2.17–2.48).

**Conclusion:**

There were large differences in health risk indicators across occupational groups, mainly between high-skilled white-collar occupations and the other occupations, with important variations also between major and sub-major occupational groups. Future health interventions should target the occupational groups identified with the highest risk for effective disease prevention.

## Background

Recent research have implied lower risk of cardiovascular disease [1, 2] and mortality [3] in white collar occupations compared to blue collar occupations. Physical activity, other lifestyle habits, physiological characteristic and social factors explains a large part of the variation between different occupational groups [4–7]. For example, white-collar occupations are reported to sit more at work and be more physically active in leisure time, while blue-collar occupations have a higher total amount of daily physical activity [4]. Moreover, risk factors such as smoking, obesity and hypertension are commonly prevalent in blue-collar occupations [5, 6], at the same time as social status and benefits are lower in blue collar occupations [7].

The sub-categorisation into white- and blue-collar occupations may mislead as these are heterogeneous groups of occupations with a diversity of work situations that could have an effect on health outcomes [8–10]. For instance, previous reports have indicated variations in overweight, smoking as well as occupational and leisure time physical activity between a larger range of occupational groups [8–10], with conflicting results of differences in cardiorespiratory fitness [8, 11]. A recent Swedish study reported large differences in diabetes type 2 incidence between the 30 most common occupations. The authors pointed at variations in underlying lifestyle related factors to possibly influence the variation in diabetes incidence between occupational groups, however had no data to study this [10]. Hence, for a clearer picture, further evaluation of differences between occupational groups regarding lifestyle-associated health risks for disease occurrence in high-powered cohorts are needed.

We aimed to identify and compare lifestyle-associated health risk indicators for common chronic diseases between different occupational groups, including a high-resolution classification of occupation, using a population-based sample of Swedish workers. We hypothesized that there would be a difference in health risk indicators not only between white- and blue-collar occupations, but also between specific major and sub-major occupational groups.

## Methods

The present study was based on the HPI Health Profile Institute cohort (HPI, Stockholm, Sweden. www.hpihealth.se), containing data from Health Profile Assessments (HPAs) carried out by employees at companies connected to occupational or other healthcare services. The HPA consists of a questionnaire including physical activity pattern, lifestyle factors and perceived health, a dialogue with a HPA coach, and a physical examination. All data were subsequently stored in the central database. The test protocol, methods used and education of HPA coaches follows a standardized procedure and has been the same since the start of HPA in the middle of the 1970s. Participation was voluntary and free of charge for the employee. Although data were available in the database since the 1980s, we based the present analyses on data from January 2014 to November 2019 to get a current analysis of the working population (*n* = 107,170). After exclusion of individuals with missing data for occupational group (*n* = 34,294) and individuals < 18 and > 75 years of age (*n* = 21), the final cross-sectional sample consisted of 72,855 participants (41% women).

### Classification of occupational groups

Occupation was reported by the participants and documented into the HPI database coded as a Swedish Standard Classification of Occupation (SSYK) [12] number. SSYK is a categorization of occupations based on the international Standard Classification of Occupation (ISCO) [13]. Each occupation is labelled and defined by a four-digit code, which refers to the job performed (defined as the tasks and duties of an employee) and the degree of qualification needed (defined as the knowledge and expertise needed to perform the tasks and duties of an occupation) for each occupation. The four-digit codes contained information on different levels; first digit defines Major group of occupation (e.g. 5 = *Service, care and shop sales*), second digit defines Sub-major group (53 = *Personal care occupations*), third digit refers to Minor group (531 = *Child minders and teacher aids*) and fourth digit Unit group (5311 = *Child care occupations*). Ten major groups of occupations were defined; 1 = *Managers*, 2 = *Professionals*, 3 = *Associate professionals*, 4 = *Administrative and customer service*, 5 = *Service, care and shop sales*, 6 = *Agricultural and forestry*, 7 = *Building and manufacturing*, 8 = *Mechanical manufacturing and transport*, 9 = *Elementary occupations* and 10 = *Military*. The first nine were included in the present analyses and *Military* were excluded due to low N. As there were further heterogeneity within these nine occupational groups, for example with regard to contact with clients/patients/students (which may induce a different psychosocial working situation) or occupational physical activity pattern [9, 14, 15], sub-major groups were identified a priori to the analyses based on this heterogeneity. Typically, contact workers were defined as occupations where the main goal is to nurse and/or educate individuals while physically active occupations where defined as workers in more physical demanding occupations than their major group. Sub-major groups *Health care* (SSYK 22) and *Education* (SSYK 23) were defined as contact workers, while *Science and engineering* (SSYK 21) and *All other professionals* (SSYK 24) were defined as non-contact workers. *Personal care occupations* (SSYK 53) was defined as contact workers, and *Service and shop sales* (SSYK 51–52) as non-contact workers. *Mechanical manufacturing* (SSYK 81–82) was defined as physically demanding and *Transport* (SSYK 83) as less physically demanding. In the tables and figures, these were labelled as a sub-code based on the Major group digit (2.1, 2.2 etc), rather than their SSYK-code. Occupational groups were aggregated into white- (Major group 1–5) and blue-collar (major group 6–9) occupations, and further by skill-level within white- and blue-collar occupations; high-skilled white-collar (major group 1–3), low-skilled white-collar (major group 4–5), high-skilled blue-collar [6, 7] and low-skilled blur-collar [8, 9, 16, 17]. Description of type and numbers of workers on Minor group level is presented in Additional file 1.

### Physical activity pattern

Exercise, physical working situation, sitting at work and sitting in leisure were self-reported through the following statements; *I exercise for the purpose of maintaining/improving my physical fitness, health and well-being …* with the alternatives Never, Sometimes, 1–2 times/week, 3–5 times/week, or At least 6 times/week; *My physical work situation …* Sitting with some movement, Physically active, Occasionally physically demanding, or Occasionally very physically demanding; *I sit at work …* and *I sit in leisure time …* Almost all the time, 75% of the time, 50% of the time, 25% of the time and Almost never.

### Physical examination indicators

Body mass and height were obtained with standard measures in light-weighing clothes, and BMI was subsequently calculated (kg·m^− 2^). Systolic and diastolic blood pressure (mmHg) were measured in the right arm using the standard auscultatory method after 20 min of seated resting. Cardiorespiratory fitness was assessed as estimated VO_2_max, expressed in ml·min^− 1^·kg^− 1^, using the submaximal Åstrand cycle test [18]. The Åstrand test has been validated against directly measured VO_2_max during treadmill running in an adult population with non-significant mean differences on group level (− 0.07 L·min^− 1^, 95% CI − 0.21 to 0.06) and with an absolute error and coefficient of variance similar to other submaximal tests (SEE = 0.48 L·min^− 1^, CV = 18.1%) [19].

### Perceived health and other lifestyle-related indicators

Perceived health and diet were self-reported through the statements *I perceive my physical and mental health as...* and *I consider my diet, regarding both meal frequency and nutritional content to be …* with the alternatives Very poor, Poor, Neither good or bad, Good, or Very good. Smoking habits and civil status derived by the statements *I smoke …* with the alternatives At least 20 cig/day, 11–19 cig/day, 1–10 cig/day, Occasionally, or Never; and *Civil status …* Living alone, Living alone with children, Living together with someone, Living together with someone and with children.

### Internal and external validity analyses

Internal validity analysis; missing data was low for exercise (0.3%), civil status (3%), smoking (0.3%), BMI (1.1%), blood pressure (1%), perceived health (0.4%) and diet (0.3%), while it was higher for cardiorespiratory fitness (22.2%), physical work situation (17.5%), sitting at work (22.4%) and sitting in leisure (22.6%). Comparing participants with missing data for the four latter variables and those included in the analyses revealed statistically significant but small differences in other central variables (Additional file 2). External validity analysis; sex- and age-distribution in major and sub-major groups in the present study were compared with national register data from 2014 to 2018 (Statistics Sweden: www.scb.se). The proportion of women in different major groups in the HPA data was similar to national register data, with only three occupational groups having more than a 5% difference; *Health professionals* (79% vs 67%), *Personal care workers* (83% vs 90%) and *Elementary occupations* (54% vs 60%) (Additional file 3). Only two occupational groups in the present data had a difference in mean age of greater than two years; *Service and sales workers* (37y vs 43y) and *Elementary occupations* (38y vs 44y).

### Statistical analysis

Chi-square test was used in the non-response analysis of internal missing data. For external validity, the proportion women and mean age was compared numerically. Independent t-tests were used to test for differences between continuous variables between high and low-skilled occupations (Table 1). Ten health risk were identified and dichotomized according to alternatives of reply or conventional cut-off values for increased health risk; No regular exercise (Never or Sometimes), Physically demanding work (Occasionally physically demanding or Occasionally very physically demanding), High sitting at work (Almost all the time or 75% of the time), High sitting in leisure (Almost all the time or 75% of the time), Perceived poor health (Very poor or poor), Perceived poor diet (Very poor or poor), Daily smoker (≥1 cig/day), Obesity (BMI > 30 kg·m^− 2^), Hypertension (diastolic BP ≥ 90 and systolic BP ≥ 140 mmHg or using self-reported blood pressure medicine) and Low cardiorespiratory fitness (estimated VO_2_max < 32 ml·min^− 1^·kg^− 1^). A total health risk indicator was derived by adding the single risk indicators, ranging from zero to seven possible risk factors for each participant (excluding physically demanding work, high sitting at work and high sitting in leisure due to high internal missing), and further dichotomized into ≥3 risk indicators or fewer for clustered risk analyses. Logistic regression modelling was used to study sex- and age-adjusted odds ratios (OR) with 95% CI confidence intervals (CI) for a) the ten different risk indicators and b) the clustered risk variable, in relation to the major and sub-major occupational groups (Managers set as reference), as well as c) the clustered risk variable in relation to grouping of occupations into blue collar and white collar, high-skilled and low-skilled (white high-skilled set as reference). Data were handled and analysed using R 3.6.3 (R Core Team, 2018) and the *Tidyverse* [20], the *jtools* [21] and the *finalfit* [22] packages.

## Results

There were significantly more men among blue-collar occupations (84% for all blue-collar occupations and 92% men in high-skilled and 74% low-skilled blue-collar workers), while there was a more equal sex-distribution in white-collar occupations (48% for all white-collar occupations and 55% in high-skilled and 32% in low-skilled white-collar occupations). In term of the separate occupational groups, there were several differences in the sex- and age-distributions (Tables 1 and 2). Regardless of being a blue- or white-collar occupations, participants with high-skilled occupations had lower BMI, lower systolic and diastolic blood pressure and higher cardiorespiratory fitness compared to participants with low-skilled occupations (all *p* < 0.001), (Table 1). There were further differences in these variables between the sub-major groups defined a priori (Table 2).

**Table 1:** Characteristics of the nine major occupational groups. In parathesis: prevalens (%) or standard deviation (SD)

	White-collar, high-skilled			White-collar, low-skilled		Blue-collar, high-skilled		Blue-collar, low-skilled		
	1	2	3	4	5	6	7	8	9	
	Managers	Professionals	Associate professionals	Administration and customer service	Service, care and shop sales	Agricultural and forestry	Building and manufacturing	Mechanical manufacturing and transport	Elementary occupations	Total
Count (% Men)	5006 (64.6%)	16,868 (46.9%)	14,513 (61.5%)	6452 (35.5%)	7961 (28.3%)	800 (63.6%)	11,267 (94.5%)	7675 (84.1%)	2313 (39.6%)	72,855 (59.2%)
Age (yrs)										
Women	46.0 (8.9)	41.7 (10.9)	41.6 (11.1)	42.8 (11.7)	42.8 (12.3)	41.0 (12.0)	37.7 (12.8)	41.3 (12.2)	45.1 (11.7)	42.4 (11.5)
Men	46.8 (9.1)	41.9 (11.2)	42.8 (11.3)	40.3 (12.6)	41.7 (12.5)	43.1 (12.9)	38.9 (13.1)	42.0 (12.6)	43.0 (13.4)	41.7 (12.2)
No relationship										
Women	176 (10.1%)	1204 (13.9%)	787 (14.4%)	676 (16.6%)	830 (14.9%)	54 (19.1%)	123 (20.3%)	201 (16.7%)	224 (16.2%)	4275 (14.7%)
Men	216 (6.9%)	1362 (17.8%)	1328 (15.3%)	554 (25.1%)	472 (21.8%)	122 (25.7%)	2342 (23.1%)	1442 (22.8%)	223 (25.0%)	8061 (19.3%)
BMI (kg·m^−2^)										
Women	25.1 (4.4)	24.8 (4.5)	25.1 (4.6)	25.7 (4.9)	27.0 (5.7)	24.5 (4.0)	25.6 (4.9)	26.4 (5.1)	27.0 (5.2)	25.6 (5.0)
Men	26.8 (3.7)	25.8 (3.8)	26.5 (4.0)	27.1 (4.6)	27.1 (4.3)	26.9 (4.7)	26.8 (4.3)	27.6 (4.7)	27.5 (4.8)	26.7 (4.2)
Systolic BP (mmHg)										
Women	120.9 (15.6)	119.8 (15.4)	120.2 (15.2)	121.7 (15.7)	123.7 (16.6)	117.7 (13.7)	119.0 (14.8)	120.9 (14.6)	125.8 (16.7)	121.2 (15.8)
Men	130.4 (14.8)	127.6 (14.1)	128.9 (14.7)	129.3 (14.9)	130.1 (15.4)	130.1 (17.4)	129.5 (15.1)	129.6 (15.2)	131.1 (15.8)	129.2 (14.9)
Diastolic BP (mmHg)										
Women	77.0 (10.3)	75.7 (10.3)	76.1 (10.3)	77.3 (10.5)	77.2 (10.7)	74.8 (9.3)	75.7 (10.5)	76.4 (10.3)	78.5 (10.2)	76.5 (10.4)
Men	81.3 (10.6)	78.9 (10.3)	80.0 (10.6)	80.1 (10.9)	80.7 (11.1)	80.0 (11.5)	79.9 (11.4)	80.3 (11.4)	81.0 (11.5)	80.0 (11.0)
VO_2_max (ml·min^−1^·kg^−1^)										
Women	36.5 (10.0)	37.3 (10.4)	36.9 (10.3)	35.3 (10.1)	33.1 (9.8)	37.8 (10.2)	36.0 (10.2)	34.4 (9.5)	31.6 (8.7)	35.7 (10.2)
Men	36.0 (9.5)	38.1 (10.3)	36.5 (10.1)	35.0 (9.9)	35.1 (9.9)	36.3 (10.5)	35.8 (9.8)	33.9 (9.3)	33.7 (9.5)	36.0 (10.0)

**Table 2:** Characteristics of the sub-major occupational groups. In parathesis: prevalens (%) or standard deviation (SD)

	2	2.1	2.2	2.3	2.4	5	5.1	5.2	8	8.1	8.2
	Professionals	Science and engineering	Health care	Education	Other professionals	Service, care and shop sales	Service and shop sales	Personal care	Mechanical manufacturing and transport	Mechanical manufacturing	Transport
Count (% Men)	16,868 (46.9%)	5005 (64.5%)	2522 (32.5%)	2460 (21.2%)	6881 (48.5%)	7961 (28.3%)	3970 (47.2%)	3991 (9.5%)	7675 (84.1%)	5654 (81.3%)	2021 (92.1%)
Age (yrs)											
Women	41.7 (10.9)	37.8 (9.6)	42.3 (11.5)	44.2 (11.0)	41.9 (10.7)	42.8 (12.3)	43.1 (12.2)	42.7 (12.3)	41.3 (12.2)	42.0 (12.1)	37.2 (12.0)
Men	41.9 (11.2)	41.3 (11.0)	43.1 (11.0)	43.5 (12.2)	42.0 (11.3)	41.7 (12.5)	42.6 (12.4)	37.5 (12.2)	42.0 (12.6)	41.8 (12.4)	42.3 (12.9)
No relationship											
Women	1204 (13.9%)	268 (15.5%)	202 (12.5%)	200 (10.6%)	534 (15.5%)	830 (14.9%)	320 (15.8%)	510 (14.4%)	201 (16.7%)	165 (15.7%)	36 (23.7%)
Men	1362 (17.8%)	556 (17.7%)	117 (15.0%)	100 (20.1%)	589 (18.1%)	472 (21.8%)	348 (19.4%)	124 (33.6%)	1442 (22.8%)	1045 (23.1%)	397 (21.9%)
BMI (kg·m^−2^)											
Women	24.8 (4.5)	23.7 (3.9)	25.0 (4.5)	25.8 (4.9)	24.7 (4.5)	27.0 (5.7)	26.6 (5.5)	27.2 (5.8)	26.4 (5.1)	26.4 (5.1)	26.5 (5.0)
Men	25.8 (3.8)	25.6 (3.6)	26.1 (3.6)	26.4 (4.4)	25.8 (3.8)	27.1 (4.3)	27.0 (4.1)	27.6 (5.1)	27.6 (4.7)	27.3 (4.5)	28.3 (5.2)
Systolic BP (mmHg)											
Women	119.8 (15.4)	116.0 (13.4)	121.0 (15.1)	123.0 (16.6)	119.4 (15.3)	123.7 (16.6)	124.4 (17.3)	123.3 (16.2)	120.9 (14.6)	121.1 (14.6)	119.0 (14.3)
Men	127.6 (14.1)	126.9 (13.5)	127.8 (13.6)	128.6 (14.4)	128.2 (14.7)	130.1 (15.4)	130.6 (15.5)	127.9 (14.6)	129.6 (15.2)	129.0 (15.1)	131.1 (15.4)
Diastolic BP (mmHg)											
Women	75.7 (10.3)	73.3 (9.3)	76.0 (10.2)	77.5 (10.8)	75.7 (10.4)	77.2 (10.7)	77.9 (11.1)	76.9 (10.5)	76.4 (10.3)	76.5 (10.3)	75.5 (10.2)
Men	78.9 (10.3)	78.3 (10.2)	79.5 (10.1)	79.3 (10.4)	79.3 (10.5)	80.7 (11.1)	81.0 (11.1)	79.0 (11.1)	80.3 (11.4)	79.8 (11.5)	81.6 (11.3)
VO_2_max (ml·min^−1^·kg^−1^)											
Women	37.3 (10.4)	40.5 (10.4)	36.4 (10.0)	34.6 (9.5)	37.6 (10.5)	33.1 (9.8)	33.7 (10.1)	32.7 (9.6)	34.4 (9.5)	34.2 (9.5)	36.1 (9.8)
Men	38.1 (10.3)	38.9 (10.4)	36.7 (9.5)	37.0 (10.4)	37.8 (10.3)	35.1 (9.9)	35.5 (10.0)	33.6 (9.5)	33.9 (9.3)	34.3 (9.4)	32.9 (9.0)

### Health risk indicators across occupational groups

**Fig. 1:**
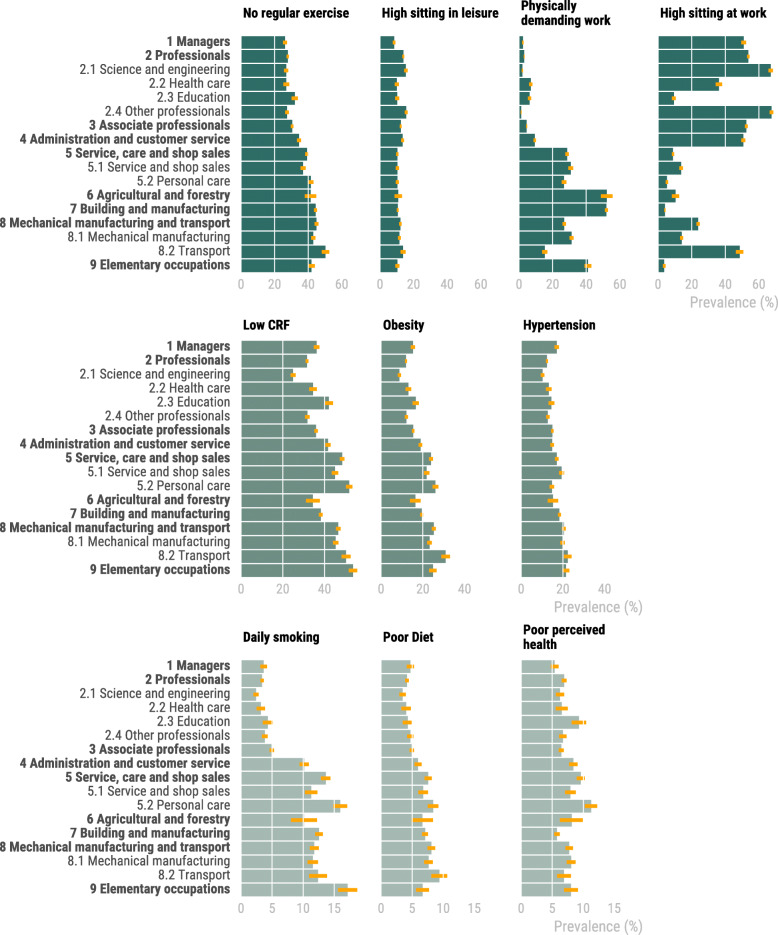
Prevalence of risk indicators in occupational groups. CRF; Cardiorespiratory fitness

Risk indicators with the highest overall prevalence were low cardiorespiratory fitness (39%) and no regular exercise (35%) (Fig. 1. Additional file 4). Risk indicators with the lowest overall prevalence were low perceived health (7%) and high sitting at leisure (12%). Multivariable adjusted analyses were performed to minimize the influence of differences in sex and age across occupational groups, although this did not materially change our findings (Table 3). With the exception of high sitting at work and in leisure, the ORs were in general lowest for white-collar high-skilled occupations, with higher OR for blue-collar occupations and low-skilled occupations. The greatest variation in OR across sub-major and major occupational groups were seen for daily smoking (OR = 0.68 to OR = 5.12), physically demanding work (OR = 0.55 to OR = 45.74) and high sitting at work (OR = 0.04 to OR = 1.86).

**Table 3:** Odds ratios and 95% CI of 10 risk indicators in 17 occupational groups

	No regularexercise	High sitting inleisure	Physicallydemanding work	High sittingat work	Low CRF	Obesity	Hypertension	Dailysmoker	Perceived poor diet	Perceived poor health
1 Managers	1 (Reference)	1 (Reference)	1 (Reference)	1 (Reference)	1 (Reference)	1 (Reference)	1 (Reference)	1 (Reference)	1 (Reference)	1 (Reference)
2 Professionals	1.18 (1.10–1.27)	1.79 (1.59–2.03)	1.32 (1.05–1.65)	1.07 (0.99–1.15)	1.09 (1.00–1.18)	0.84 (0.77–0.92)	1.01 (0.92–1.11)	0.90 (0.76–1.07)	0.79 (0.68–0.92)	1.25 (1.09–1.44)
2.1 Science and engineering	1.12 (1.02–1.23)	1.83 (1.58–2.11)	0.81 (0.59–1.10)	1.82 (1.66–2.00)	0.86 (0.78–0.96)	0.61 (0.54–0.70)	0.83 (0.73–0.94)	0.68 (0.54–0.86)	0.59 (0.48–0.72)	1.20 (1.02–1.42)
2.2 Health care	1.15 (1.03–1.28)	1.34 (1.11–1.63)	3.26 (2.48–4.27)	0.49 (0.43–0.55)	1.19 (1.06–1.34)	0.95 (0.83–1.10)	1.13 (0.98–1.31)	0.81 (0.62–1.06)	0.83 (0.65–1.05)	1.13 (0.93–1.39)
2.3 Education	1.48 (1.32–1.64)	1.48 (1.23–1.78)	2.97 (2.27–3.89)	0.09 (0.08–0.11)	1.54 (1.37–1.74)	1.24 (1.09–1.42)	1.24 (1.07–1.43)	1.07 (0.83–1.36)	0.97 (0.76–1.23)	1.60 (1.33–1.93)
2.4 Other professionals	1.15 (1.06–1.25)	2.02 (1.76–2.31)	0.55 (0.40–0.74)	1.86 (1.71–2.03)	1.09 (1.00–1.19)	0.86 (0.77–0.96)	1.04 (0.93–1.15)	1.03 (0.85–1.25)	0.90 (0.76–1.08)	1.22 (1.05–1.43)
3 Associate professionals	1.30 (1.21–1.40)	1.45 (1.28–1.65)	1.97 (1.58–2.46)	1.01 (0.94–1.08)	1.32 (1.22–1.43)	1.10 (1.01–1.21)	1.11 (1.01–1.22)	1.40 (1.19–1.65)	0.92 (0.79–1.07)	1.22 (1.06–1.40)
4 Administration and customer service	1.64 (1.51–1.79)	1.81 (1.57–2.08)	4.43 (3.54–5.55)	0.96 (0.88–1.04)	1.75 (1.59–1.92)	1.47 (1.33–1.63)	1.33 (1.20–1.49)	2.80 (2.36–3.32)	1.19 (1.00–1.40)	1.49 (1.28–1.74)
5 Service, care and shop sales	2.02 (1.87–2.19)	1.38 (1.20–1.58)	17.30 (14.00–21.37)	0.09 (0.08–0.10)	2.29 (2.10–2.51)	1.98 (1.80–2.18)	1.58 (1.42–1.75)	3.80 (3.23–4.48)	1.61 (1.37–1.88)	1.68 (1.45–1.94)
5.1 Service and shop sales	1.78 (1.63–1.95)	1.22 (1.03–1.45)	18.92 (15.22–23.52)	0.14 (0.13–0.16)	1.96 (1.76–2.17)	1.72 (1.54–1.92)	1.62 (1.44–1.82)	3.27 (2.73–3.91)	1.36 (1.13–1.63)	1.45 (1.23–1.72)
5.2 Personal care	2.33 (2.13–2.56)	1.50 (1.28–1.76)	16.43 (13.20–20.46)	0.05 (0.04–0.05)	2.76 (2.48–3.07)	2.35 (2.10–2.62)	1.55 (1.37–1.76)	4.40 (3.69–5.23)	1.94 (1.62–2.32)	1.93 (1.64–2.26)
6 Agricultural and forestry	2.10 (1.80–2.46)	1.28 (0.94–1.74)	45.74 (35.39–59.12)	0.10 (0.08–0.14)	1.21 (1.01–1.46)	1.19 (0.97–1.46)	1.04 (0.83–1.30)	3.06 (2.33–4.02)	1.24 (0.91–1.68)	1.58 (1.19–2.09)
7 Building and manufacturing	2.37 (2.20–2.56)	1.00 (0.88–1.15)	42.67 (34.64–52.57)	0.04 (0.03–0.04)	1.88 (1.73–2.05)	1.54 (1.40–1.69)	1.50 (1.37–1.65)	4.45 (3.79–5.22)	1.08 (0.93–1.26)	1.24 (1.06–1.43)
8 Mechanical manufacturing and transport	2.37 (2.19–2.57)	1.28 (1.12–1.47)	14.92 (12.08–18.43)	0.28 (0.25–0.30)	2.26 (2.07–2.46)	2.04 (1.85–2.24)	1.49 (1.35–1.64)	3.91 (3.31–4.61)	1.42 (1.21–1.66)	1.61 (1.38–1.87)
8.1 Mechanical manufacturing	2.19 (2.02–2.38)	1.22 (1.05–1.41)	18.38 (14.86–22.75)	0.15 (0.14–0.17)	2.10 (1.91–2.30)	1.81 (1.64–2.00)	1.45 (1.31–1.61)	3.76 (3.17–4.45)	1.33 (1.13–1.57)	1.65 (1.41–1.93)
8.2 Transport	2.89 (2.59–3.22)	1.44 (1.21–1.73)	7.18 (5.64–9.15)	0.90 (0.80–1.01)	2.71 (2.37–3.09)	2.65 (2.34–3.00)	1.57 (1.37–1.81)	4.22 (3.45–5.15)	1.61 (1.32–1.97)	1.45 (1.17–1.79)
9 Elementary occupations	2.18 (1.96–2.42)	1.34 (1.11–1.62)	30.10 (24.07–37.64)	0.04 (0.03–0.05)	2.64 (2.33–2.99)	1.98 (1.75–2.24)	1.74 (1.52–1.99)	5.12 (4.26–6.16)	1.41 (1.15–1.75)	1.42 (1.17–1.73)

All analyses were adjusted for age and sex

Within major occupational group *Professionals* [2], non-contact workers in sub-major group *Science and engineering* (2.1) had the lowest OR among the sub-major groups (except for high sitting at work and in leisure), and contact workers in sub-major group had the highest OR (except for high sitting at work and in leisure). Similar adverse OR profile were seen in sub-major group *Personal care* (5.2, contact workers) and *Transport* (8.2, less physically demanding occupations), compared to their counterparts. On the contrary, *Agricultural and forestry* had a more beneficial OR profile compared to other high-skilled blue-collar occupations. In the physiological variables, obesity and cardiorespiratory fitness there was a general pattern of higher OR in low-skilled compared to high-skilled occupations.

### Clustering of risk across occupational categories

**Fig. 2:**
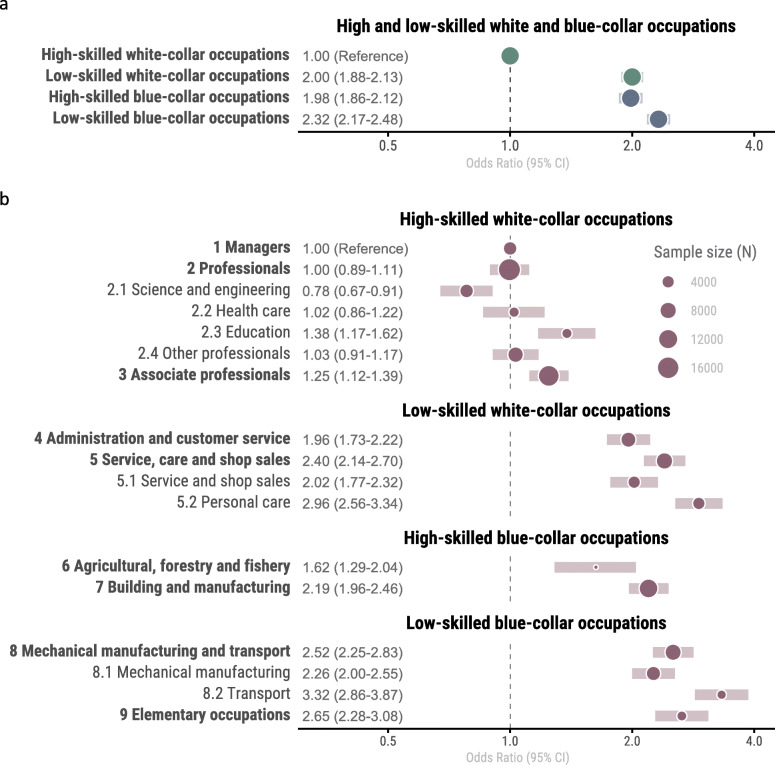
**a** OR (95% CI) for clustering of risk indicators in relation to aggregation of occupations into white collar, blue collar, high-skilled and low-skilled occupations. **b** OR (95% CI) for clustering of risk indicators in relation to major and sub-major occupational groups

OR for clustering of health risk indicators was 1.80 (95% CI; 1.71–1.90) in blue-collar compared to white-collar occupations (Fig. 2a). The clustered risk was similar in low-skilled white-collar (2.00; 1.88–2.13) and high-skilled blue-collar (1.98; 1.86–2.12) occupations compared to high-skilled white-collar occupations (reference), while low-skilled blue-collar occupations had a further higher odds ratio compared to the other groups (2.32; 2.17–2.48).

Odds ratio for clustered risk varied considerably between and within major and sub-major occupational groups (Additional file 5 and Fig. 2b). While *Professionals* (major group 2) had similar OR as *Managers* (major group 1, reference), the sub-major group *Science and engineering* (2.1) had significantly lower OR (0.78; 0.67–0.91) and sub-major group *Education* (2.3) had significantly higher OR (1.38; 1.17–1.62), than major group *Professionals* [2]. Low-skilled white-collar occupations had two- to three-fold increased OR for clustered risk compared to reference, with *Personal care* occupations (5.2) having the highest OR (2.96; 2.56–3.34). *Agricultural and forestry* (major group 6) had the lowest OR for clustered risk among blue-collar occupations (1.62; 1.29–2.04), albeit with a smaller sample size than in other occupations. *Transport* and *Elementary occupations* had the highest OR (3.32; 2.86–3.87 and 2.65; 2.28–3.08) for clustered risk in blue-collar occupations. In further sensitivity analysis, using ≥5 risk indicators as cut-off did not alter the odds ratios across categories.

## Discussion

The main findings were large differences in health risk indicators across a wide range of occupations, including in-between different major and sub-major occupational groups. Moreover, high-skilled occupations had in general a more beneficial risk profile compared to low-skilled occupations, regardless of being white- or blue-collar, with a two- to three-fold higher clustered health risk. The sub-major occupations *Education, Personal care* and *Transport* had in general a more adverse risk profile compared occupations of the same major occupational group. On the contrary, *Science and engineering* as well as *Agricultural and forestry* had a more beneficial risk profile.

### Single risk indicators in occupational groups

We are not aware of any other large-scale investigation examining a combination of several health risk indicators in different high-resolution occupational groups. Though, the physical activity patterns at work and in leisure time in the present study are partly similar to previous reports in both major occupational groups and in some sub-major groups [8, 9, 23]. For example, white collar/professional occupations are consistently reported to have a higher amount of leisure time moderate-to-vigorous physical activity compare to blue collar workers [4, 23], with less differences in leisure time sitting [24, 25]. On the contrary, blue-collar occupations have typically a more physically demanding work and less occupational sitting than white collar workers [8, 9]. However, while white-collar workers in the present study reported higher sitting at work compared to leisure time, previously found and described as “compensation effect” [25], no similar compensation pattern was seen for blue-collar workers. Additionally, we found some exceptions to the above patterns for sub-major occupational groups within white- and blue-collar occupations. Sub-major occupational group *Education* and *Health care* had significantly lower amount of sitting at work, and *Education* lower levels of regular exercise, compared to other high-skilled white-collar workers. Similarly, *Personal care* workers had lower levels of regular exercise than the general low-skilled white-collar worker. Among blue-collar workers, *Transport* workers had significantly higher sitting at work and less regular exercise in leisure, which has also previously been reported [23].

Interestingly, we found that low cardiorespiratory fitness mainly varied between high- and low-skilled occupations (low-skilled having poorer cardiorespiratory fitness than high-skilled), with less variation between the average blue- and white-collar worker, respectively. Our findings are not consistent with U.S. and Swiss data, where blue collar workers had higher cardiorespiratory fitness compared to white-collar and service personnel [11] and no differences in fitness was found between major occupational groups [8]. However, a previous Swedish study reported low physical fitness for male conscripts whose future occupation would be motor vehicle drivers, agricultural and other mobile plant operators [10].

Obesity prevalence showed similar pattern across occupational groups as low CRF. One reason for this could be that these two indicators share the weight characteristic in their formulae, although they can still be seen as separate risk indicators [26]. Previous studies have reported similar findings, with higher obesity prevalence for women and men with low social position occupations [10, 27] Also hypertension shared a similar pattern as low cardiorespiratory fitness and obesity, which is in line with previous studies reporting higher risk of hypertension in blue-collar workers in comparison to white-collar workers [5, 28, 29]. This may partly be attributed to that excess body fat is a strong predictor of hypertension, but also with covariation with other risk indicators such as smoking [30].

Earlier studies have reported higher smoking in blue collar occupations [31, 32]. In the present study, we saw a clear distinction between high-skilled white-collar workers and all other occupational groups, where in general 5% or less were daily smokers in the previous group compared to between 10 and 15% in the latter. On the contrary to daily smoking, perceived poor health varied less between white- and blue-collar workers, however, with general lower risk of perceived poor health in high skilled white-collar occupations compared to all other occupational groups. This is in line with a previous study from Germany, where professionals (high-skilled white-collar workers) had a lower risk of poor health, and service and unskilled manual workers had the highest risk [33]. However, there were further variations within white and blue-collar occupation in the present study. Sub-major contact-worker groups *Education* and *Personal Care* having considerably higher risk compared to their counterparts while the major occupational groups *Mangers* and *Building and manufacturing* had the lowest risk of perceived poor health in white and blue-collar workers respectively.

### Clustering of risk indicators in occupational groups

High-skilled white-collar workers had in general the lowest clustered risk. Interestingly, low-skilled white-collar workers (*administration and customer service* and *service, care and shop sales*) had similar increased risk as blue-collar workers (two-fold compared to high-skilled white-collar). Only *agricultural and forestry workers* did not have a significant higher risk compared to the general high-skilled white-collar occupation. On the contrary, *Education*, *Personal care* and *Transport* workers did all have a generally higher risk than their white−/blue-collar and low−/high-skilled counterparts. In a previous study on 6282 employed Americans, prevalence of optimal composite clinical and behavioural cardiovascular health scores were in general low, with large variation between major occupational groups [34]. Sales and low status office workers had a low prevalence of optimal total cardiovascular health score, and especially computer and healthcare support workers had a low prevalence of optimal behavioural health. Accumulation of multiple healthy or unhealthy lifestyle indicators is shown to strongly associate with multi-morbidity, disability-adjusted life-years gained and years lived without a chronic disease [35–37]. Hence, the previous reports of variation in diabetes type 2 prevalence [10] and mortality [15] in Swedish workers may partly be associated with underlying variation in lifestyle risk behaviours similar to the ones in the present study. Hence, specifically targeting the major and sub-major occupational groups with highest clustering of risk indicators is a crucial step for disease prevention.

### Sex differences

Many articles report larger socioeconomic differences in men in comparison to women [14]. These studies often uses absolute measures of socioeconomic differences where the magnitude of the differences between groups is retained. However when using relative difference of socioeconomic inequality, that is the ratio of the difference between the groups, there seems not to be any significant differences [14]. In the present study, we found only small sex-differences in clustered risk between men and women (Additional file 5). Albeit, when studying prevalence by sex in all 10 risk indicators, hypertension stood out with a large prevalence difference between men and women across occupational groups (absolute difference) at the same time as daily smoking was higher in low-skilled women (relative difference).

Occupations with higher percentage of contact workers has been theorized to be psychologically heavier [14, 38]. In the present study, the majority of workers in these occupations was female workers. For example, the female dominated *Education* and *Personal care* occupations had higher clustering of risk indicators than the other occupations in each major group. We also saw that occupations with large differences in amount of women and men generally had a higher risk of clustering of risk health factors, which also is in concordance with a Swedish report on mortality in different occupational groups [15].

### A potential physical activity paradox

This study is partly in line with the proposed “Physical activity paradox” [39]. It suggests that there are contrasting health outcomes from occupational physical activity and leisure time physical activity, whereas occupational physical activity is detrimental for health while leisure time physical activity is beneficial for health. In the present study, occupations with a higher occupational physical activity had higher clustering of risk indicators. However, *Transport workers*, without a high amount of physically demanding work had among the highest clustered risk. This indicates that other health indicators related to socioeconomic status or lifestyle attributes shared by these occupations could partly confound the physical activity paradox. For example, Smith et al. [40] showed a higher risk of incident cardiovascular disease for people with standing occupations compared to sitting occupations. However, the relationship weakened and became non-significant after adjusting for educational level and variables strongly linked to socio-economic status. This itself is a paradox that could be further evaluated with prospective multi-adjusted models within and between high resolution occupational groups on future health outcomes such as sickness absence, cardiovascular disease, cancer and mortality. One suggested approach to influence the heterogeneity of the physical activity pattern in different occupational groups tested right now is “the Goldilocks Principle” [41]. It is aiming at designing physical activity at work to be “just right” for better health, with a balance between physical activity and recovery at work in focus. This balance should be achievable trough assessing intensity, duration, frequency of different postures and movements adapted to the specific demands of different occupations.

### Strengths and limitations

Strengths of this study include the large cohort of women and men from a wide variety of occupations, with high internal and external validity. Moreover, cardiorespiratory fitness was estimated using exercise testing, as were presences of obesity and hypertension. Limitations include its cross-sectional design, which prohibits causal inferences to future disease incidence. Moreover, a limitation is the self-report data for exercise, sitting in leisure and at work, as well as the lack of validation of the exercise question. However, questionnaires with categorical answer modes as used in the present study have been reported to provide superior validity compared to open answer modes for physical activity level [42]. A factor that could have lessened the differences in indicators between occupational groups are the accuracy of the classification into occupational groups, where a more accurate classification would likely strengthen many of the differences reported in the present study. Also, in the present study there were no differences in cardiorespiratory fitness between sexes which is not in concordance with the largest cohort of directly measured VO^2^max in the Nordic countries [43].

## Conclusion

The main findings of the present study were large differences in health risk indicators across a wide range of occupations, including in-between different major and sub-major occupational groups. High-skilled occupations had in general a more beneficial risk profile compared to low-skilled occupations, regardless of being white- or blue-collar, with a two- to three-fold higher clustered health risk. Differences were also found between major groups and their subgroups. The present findings provide guidance on which occupational groups that should be targeted in future health interventions. Prospective studies including both health risk indicators and longitudinal outcomes including chronic disease incidence are needed to further clarify the role of physical activity pattern and lifestyle in occupational health disparities.

## Supplementary Information

**Additional file 1.** a. Proportions of workers in minor occupational groups within major occupational groups 1 to 5 according to SSYK. (referred to as white collar occupations). b. Proportions of workers in minor occupational groups within major occupational groups 6 to 9 according to SSYK (referred to as blue collar occupations).

**Additional file 2.** Mean age, women (%), no regular exercise (%), obesity (%) and daily smokers (%) in individuals with non-missing vs individuals with missing data for the variables variables cardiorespiratory fitness, physical work situation, sitting at work and sitting in leisure.

**Additional file 3.** Percent women and mean age in major and sub-major occupational groups according to national register* (SCB) and the present study (HPB) data.

**Additional file 4.** Sex specific prevalence of risk indicators in major and sub-major occupational groups.

**Additional file 5.** Sex specific odds ratio (95% CI) for clustering of health risk indicators in relation to major and sub-major occupational groups.

## Data Availability

The data underlying the findings in our study are not publicly available because the original approval by the regional ethic’s board (Stockholm Ethics Review Board, Dnr 2015/1864–31/2 and 2016/9–32) and the informed consent from the subjects participating in the studies did not include such a direct, free access. If a reader wants access to the data underlying the present article, please contact the HPI Health Profile Institute at support@hpihealth.se.

## Abbreviations

SSYK: Swedish Standard Classification of Occupation
ISCO: International Standard Classification of Occupation
OR: Odds Ratio
