# Supplementary material for: Lifestyle-associated health risk indicators across a wide range of occupational groups: a cross-sectional analysis in 72,855 workers
Source: BMC Public Health. 2020 Nov 4;20:1656. doi: 10.1186/s12889-020-09755-6 (PMC7641800; doi:10.1186/s12889-020-09755-6)
Supplement: Supplementary file 1 — Additional file 1. a. Proportions of workers in minor occupational groups within major occupational groups 1 to 5 according to SSYK. (referred to as white collar occupations). b. Proportions of workers in minor occupational groups within major occupational groups 6 to 9 according to SSYK (referred to as blue collar occupations). [file 12889_2020_9755_MOESM1_ESM.zip › Additional_file_1bR1.pdf]

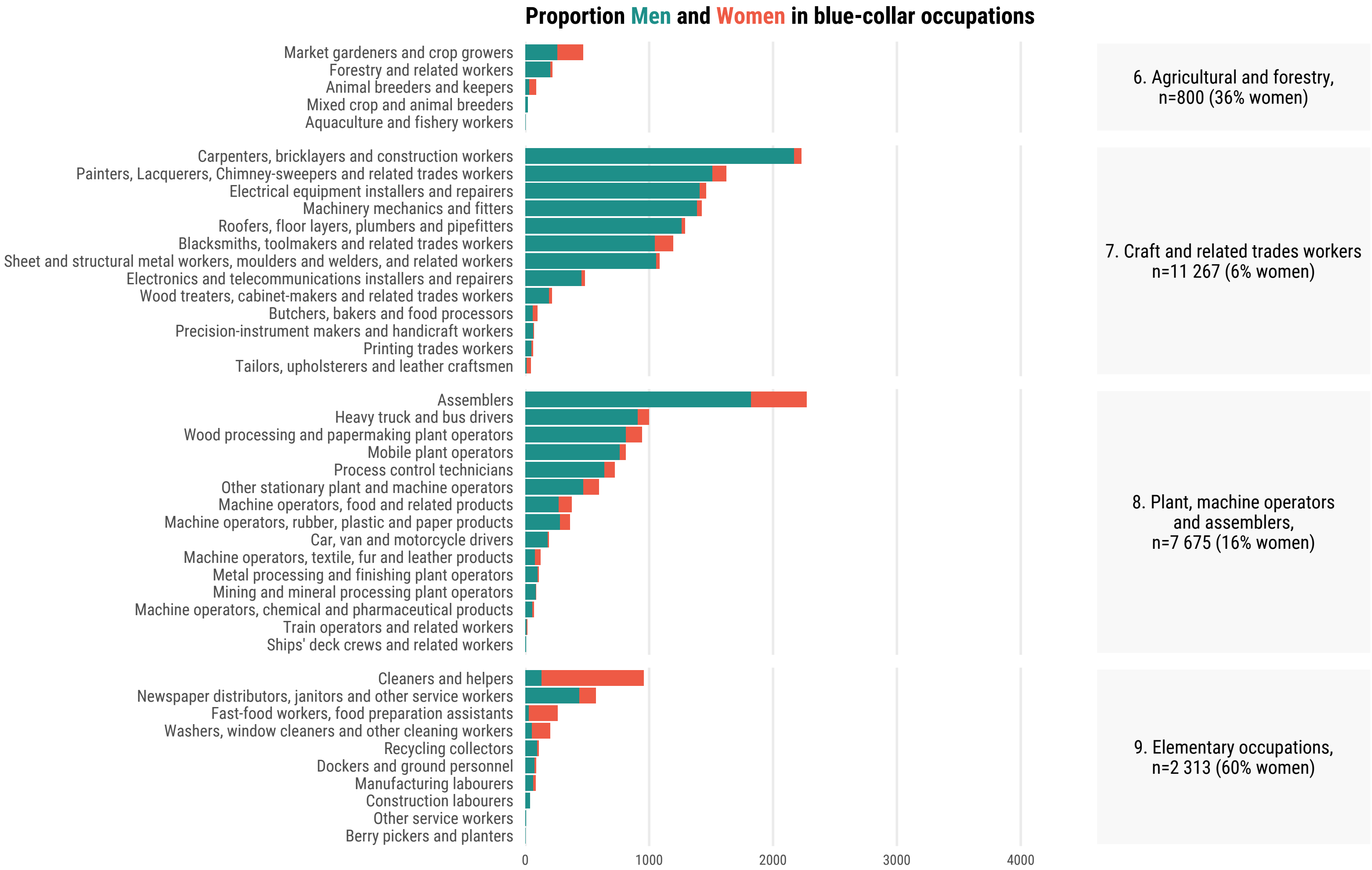

Additional file 1b. Proportions of workers in minor occupational groups within major occupational groups 6 to 9 according to SSYK (referred to as blue collar occupations).
