# Supplementary material for: Lifestyle-associated health risk indicators across a wide range of occupational groups: a cross-sectional analysis in 72,855 workers
Source: BMC Public Health. 2020 Nov 4;20:1656. doi: 10.1186/s12889-020-09755-6 (PMC7641800; doi:10.1186/s12889-020-09755-6)
Supplement: Supplementary file 2 — Additional file 2. Mean age, women (%), no regular exercise (%), obesity (%) and daily smokers (%) in individuals with non-missing vs individuals with missing data for the variables variables cardiorespiratory fitness, physical work situation, sitting at work and sitting in leisure. [file 12889_2020_9755_MOESM2_ESM.pdf]

Internal validity

Additional file 2. Mean age, women (%), no regular exercise (%), obesity (%) and daily smokers (%) in individuals with non-missing vs individuals with missing data for the variables variables cardiorespiratory fitness, physical work situation, sitting at work and sitting in leisure.

	Non-missing	Missing	Significance
**Mean age (SD):**			
Cardiorespiratory fitness	42 (12)	45 (13)	<0.001
Physical work situation	42 (12)	42 (12)	<0.001
Sitting at work	42 (12)	42 (12)	<0.001
Sitting in leisure	42 (12)	43 (12)	<0.001
**Women (%):**			
Cardiorespiratory fitness	41	39	<0.001
Physical work situation	41	40	0.006
Sitting at work	41	40	0.071
Sitting in leisure	41	40	0.01
**No regular exercise (%):**			
Cardiorespiratory fitness	34	39	<0.001
Physical work situation	35	35	<0.001
Sitting at work	35	36	<0.001
Sitting in leisure	35	36	<0.001
**Obesity (%):**			
Cardiorespiratory fitness	16	23	<0.001
Physical work situation	18	16	<0.001
Sitting at work	18	16	<0.001
Sitting in leisure	18	16	<0.001
**Daily smoking (%):**			
Cardiorespiratory fitness	8	10	<0.001
Physical work situation	8	9	<0.001
Sitting at work	8	9	<0.001
Sitting in leisure	8	9	<0.001
