# Supplementary material for: Lifestyle-associated health risk indicators across a wide range of occupational groups: a cross-sectional analysis in 72,855 workers
Source: BMC Public Health. 2020 Nov 4;20:1656. doi: 10.1186/s12889-020-09755-6 (PMC7641800; doi:10.1186/s12889-020-09755-6)
Supplement: Supplementary file 3 — Additional file 3. Percent women and mean age in major and sub-major occupational groups according to national register* (SCB) and the present study (HPB) data. [file 12889_2020_9755_MOESM3_ESM.pdf]

External validity

Additional file 3. Percent women and mean age in major and sub-major occupational groups according to national register* (SCB) and the present study (HPB) data.

	% Women		Mean Age	
	SCB	HPB	SCB	HPB
1 Managers	38	35	47	47
2.1 Science and engineering	32	36	42	40
2.2 Health care	79	67	44	43
2.3 Education	73	79	44	44
2.4 Other professionals	51	51	43	42
3 Associate professionals	41	38	42	42
4 Administration and customer service	62	65	40	42
5.1 Service and shop sales	54	53	37	43
5.2 Personal care	83	90	43	42
6 Agricultural and forestry	31	36	41	42
7 Building and manufacturing	6	6	41	39
8.1 Mechanical manufacturing	22	19	41	42
8.2 Transport	9	8	42	42
9 Elementary occupations	54	60	38	44

* Statistics Sweden, www.scb.se
