# Supplementary material for: Lifestyle-associated health risk indicators across a wide range of occupational groups: a cross-sectional analysis in 72,855 workers
Source: BMC Public Health. 2020 Nov 4;20:1656. doi: 10.1186/s12889-020-09755-6 (PMC7641800; doi:10.1186/s12889-020-09755-6)
Supplement: Supplementary file 5 — Additional file 5. Sex specific odds ratio (95% CI) for clustering of health risk indicators in relation to major and sub-major occupational groups. [file 12889_2020_9755_MOESM5_ESM.pdf]

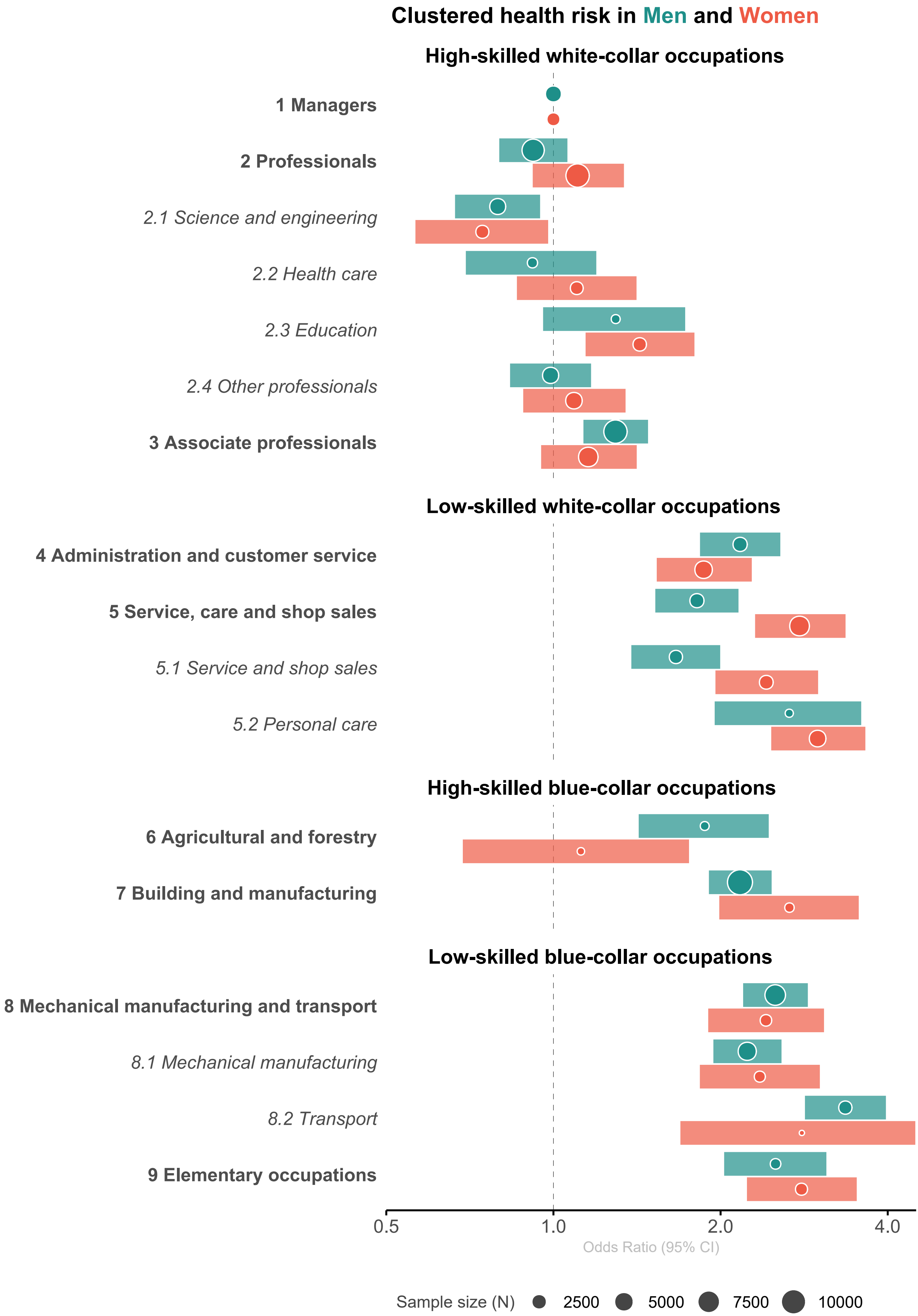

Additional file 5. Sex specific odds ratio (95% CI) for clustering of health risk indicators in relation to major and sub-major occupational groups.
